# Biomimetic 3D Hydrogels with Aligned Topography for Neural Tissue Engineering

**DOI:** 10.3390/polym16243556

**Published:** 2024-12-20

**Authors:** Liza J. Severs, Anjali Katta, Lindsay N. Cates, Dane M. Dewees, Riana T. Hoagland, Philip J. Horner, Christoph P. Hofstetter, Zin Z. Khaing

**Affiliations:** 1 Department of Physiology and Biophysics, The University of Washington, Seattle, WA 98109, USA; lkobelt@uw.edu; 2 Department of Neurological Surgery, The University of Washington, Seattle, WA 98109, USA; akatta@uw.edu (A.K.); lncates@uw.edu (L.N.C.); dane.dewees@gmail.com (D.M.D.); rianahoagland@gmail.com (R.T.H.); chh9045@uw.edu (C.P.H.); 3 Department of Neurosurgery, Houston Methodist Research Institute, Houston, TX 98109, USA; pjhorner@houstonmethodist.org

**Keywords:** anisotropic hydrogels, injectable hydrogels, neural repair, alignment, 3D hydrogels

## Abstract

Spinal cord trauma leads to the destruction of the highly organized cytoarchitecture that carries information along the axis of the spinal column. Currently, there are no clinically accepted strategies that can help regenerate severed axons after spinal cord injury (SCI). Hydrogels are soft biomaterials with high water content that are widely used as scaffolds to interface with the central nervous system (CNS). Here, we examine a simple and reproducible method that results in consistently aligned fibrils within 3D matrices using thermally gelling biomimetic polymers. A collagen type I (Col)-based thermally gelling hydrogel system was used in combination with two other native extracellular matrix proteins: laminin I (LN) and hyaluronic acid (HA). Gelling kinetics for all gel types (Col, Col LN, Col HA) showed that at 37 °C, all three hydrogels formed gels consistently. A method of aspiration and ejection was used to produce Col-based hydrogels containing aligned fibrils. In vitro, embryonic spinal cord neurons survived and produced processes aligned to collagen fibrils. Next, we implanted either non-aligned or aligned hydrogels after a bilateral dorsal hemisection of the thoracic spinal cord at T7/T8. Pan neuronal antibody-positive fibrils were found within all implants; aligned hydrogels supported neurite growth along the parallel direction of the implanted hydrogels. Combined, our in vitro and in vivo data indicate that thermally gelling biomimetic hydrogels can produce aligned matrices through a method of aspiration and ejection, and this presents a novel platform for regenerative therapies for the CNS.

## 1. Introduction

Spinal cord trauma leads to the destruction of both cells and fiber tracks that carry information in a bidirectional manner. Currently, there are no effective clinical strategies to regenerate axons after spinal cord injury (SCI). Neurons within the adult mammalian spinal cord have limited intrinsic capabilities for regeneration [1,2,3,4]. Furthermore, the regeneration of severed axons is inhibited by multiple factors, such as insufficient trophic factors [5] and inhibitory components within the glial scar and myelin debris [6,7]. Many promising experimental strategies are currently being developed to enhance reconnection and rewiring for functional recovery. These include the use of stem cells from various sources [8,9,10,11,12,13,14,15], growth factors [16,17,18], inhibiting the inhibitors of regeneration [19,20,21,22,23,24,25,26,27], engineered biomaterials [28,29], and combinations of some or all of these factors [30,31,32,33,34,35]. It has become increasingly clear that the inclusion of an optimal biomaterial with the correct physical properties to support exogenous cell growth and interact with the host spinal tissue is essential for developing a successful therapeutic strategy to support regeneration after SCI.

Soft hydrogels made from naturally derived polymers have emerged as effective therapeutic candidates because they are biocompatible and have similar mechanical properties to that of spinal cord tissue [36,37,38,39,40]. Indeed, there have been several hydrogel formulations proposed for spinal cord injury repair [41,42,43,44,45,46,47]. These include natural-based polymers and conductive and synthetic polymers. The proposed hydrogels for SCI repair strategies include single-component hydrogels and multiple-component hydrogels, like our composite hydrogels, but also ones with added biomolecules such as growth factors (readers are referred to the following excellent review articles: [6,48,49,50]). Interestingly, neurons in vitro and in vivo respond well to aligned topography during growth and regeneration. However, most hydrogels are amorphous, isotropic, and lack topographical cues. The development of polymers with consistent biomimetic topographies that promote cell growth and are easily manipulated has been challenging. Here, we report the development of a biomimetic injectable 3D hydrogel platform with aligned topography for repair after SCI.

Naturally occurring extracellular matrix (ECM) components, including collagen, have been used successfully in experimental SCI treatment strategies, as they mimic the in vivo conditions and are native to the body [29,51,52,53]. Using natural ECM components, most of which are evolutionarily conserved between different species, can also aid in limiting issues with local inflammation and immunological rejection once in vivo [54,55]. Here, we have developed biomimetic hydrogels containing collagen type I (Col), laminin (LN), and hyaluronic acid (HA). Individual triple helices, or tropocollagen, are assembled to form each collagen fibril and are fundamental to the strength of collagen in the body. Different types of collagens are characterized by the presence of segments interrupting this helix and result in variations in their three-dimensional structure [56]. Since collagen innately forms elongated fibrils, we used a simple approach combining aspiration and ejection to produce aligned scaffolding proteins that can direct neurite outgrowth. The collagen gelation time, fibril size, and chemical interactions are easily altered by factors such as pH, ionic strength, and temperature [57]. Therefore, Col I is an excellent candidate for use as a biomimetic scaffold in the spinal cord. We have also included co-gels containing LN and HA into the Col hydrogel platform as both ECM molecules are present within the spinal cord during development [58,59,60,61].

Previously, we have shown that a biomimetic 3D hydrogel scaffold (Col LN HA) without aligned topography can enhance functional recovery after a contusion SCI model in rodents [47]. Our goal for this study was to create anisotropic hydrogels (Col-based) with aligned topography utilizing this biomimetic 3D hydrogel platform. We report here that thermally gelling biomimetic 3D hydrogels can be used to produce aligned topography and provide a natural substrate to support axonal growth and alignment of primary embryonic spinal neurons in vitro. In addition, implantation of aligned hydrogels following a T7/T8 bilateral thoracic spinal cord hemisection resulted in an influx of host cells and neurite growth into both non-aligned and aligned hydrogel implants. Directionality analysis showed that neurites within the aligned implants are significantly more aligned compared to those in non-aligned hydrogels. Combined, our data support the use of natural polymer-based biomimetic hydrogels with aligned morphology to support and direct growth and regeneration in vivo.

## 2. Materials and Methods

All materials and reagents were obtained from Sigma-Aldrich unless otherwise noted.

### 2.1. Hydrogel Synthesis

**Figure 1:**
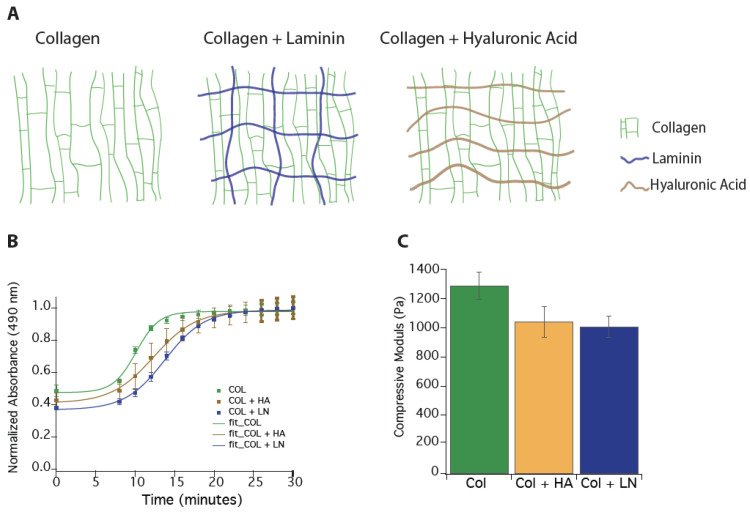
Thermally gelling collagen-based hydrogels. (**A**) Schematic of collagen type I (Col)-based thermally hydrogels. (**B**) Thermal gelation kinetics for Col, Col hyaluronic acid (HA), and Col laminin I (LN) hydrogels. Col solution forms gels faster than Col HA and Col LN solutions at 37 °C. (**C**) Bulk compressive moduli of Col (*N* = 15), Col HA (*N* = 15), and Col LN Col (*N* = 15) hydrogels.

Hydrogels were synthesized from thermally gelling rat-tail Type I collagen (Col, Corning, Corning NY, USA, CB354249, Purity > 90%), mouse laminin I (LN, Travigen, Gaithersburg, MD, USA), and high-molecular-weight (>1500 kDa) bacterial-synthesized hyaluronic acid (HA, Sigma-Aldrich, St. Louis, MO, USA, 53747, pH 7 at 20 °C) (Figure 1). HA was dissolved in distilled water at 15 mg/mL concentration to create a stock solution overnight, sterilized (using a 0.5-micron filter), and stored at 4 °C until use. First, a Col stock solution was made by diluting the stock Col solution with 0.2% acetic acid to a concentration of 6 mg/mL. A Col gel solution was made by combining the Col stock solution with 10× phosphate-buffered saline (PBS) and (4-(2-hydroxyethyl)-1-piperazineethanesulfonic acid (HEPES) buffer at an 8:1:1 ratio to obtain a final concentration of 3 mg/mL. Additionally, a pre-gel solution for Col/HA and Col/LN was produced using a final concentration of 1.5 mg/mL of either HA or LN solutions. All pre-gel solutions were made on ice to prevent premature gelation.

### 2.2. Gelation

To assess the time for gelation at 37 °C, absorbance at 490 nm was measured for 100 µL of pre-gel solutions of Col (*N* = 9), Col HA (*N* = 7), and Col LN (*N* = 4) in a 96-well plate using a Synergy HT microplate reader (BioTek, Winooski, VT). The well plate was transferred from ice immediately to the pre-warmed (37 °C) plate reader. Absorbance readings were recorded every 2 min for 30 min. A solution of Dulbecco’s Modified Eagle’s medium (DMEM) + HEPES, diluted in PBS at the same concentration as in the hydrogels, was used as a negative control. Data were normalized to the initial readings, and the time to reach 50% and 95% of the maximum turbidity, t_50_ and t_95_, respectively, were calculated. This experiment was repeated three times to ensure consistent results.

### 2.3. Compressive Modulus

The mechanical characterization of hydrogels was performed to verify that the properties are within range of the mechanical properties of native neural tissue. One hundred twenty microliters of gel solution were placed into cylindrical molds 8 mm in diameter by 2 mm deep (Grace Biolabs, Bend, OR, USA) to create gels with defined geometry. The gels were allowed to equilibrate at 20 °C in PBS for one hour prior to testing to prevent changes in mechanical properties in response to decreasing temperature during the experiment.

Bulk compressive moduli were determined using an Instron mechanical testing machine (Model 3345, Instron, Norwood, MA, USA) for the three different hydrogel compositions. Cylindrical samples were compressed at a rate of 0.1 mm/s to at least 60% strain. Moduli were calculated as the slope of the stress versus strain curve in the linear region within the first 20% of the strain [62]. The total sample size per group was *N* = 15. Hydrogels were excluded from the study if they were torn or damaged prior to or during transfer to the Instron. Data from this process were compiled to determine the average compressive modulus of each hydrogel composition.

### 2.4. Fabrication of Aligned Hydrogels

**Figure 2:**
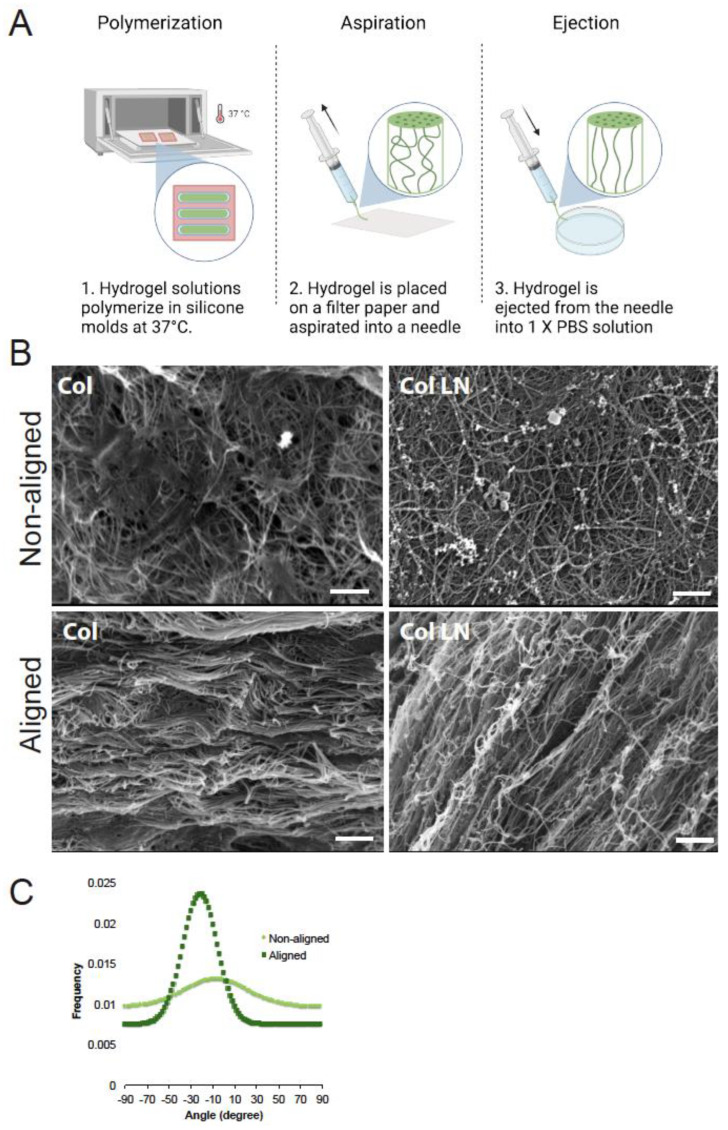
Production of anisotropic aligned hydrogels. (**A**) A schematic of gelation and aligned gel production. Cold solutions were placed into molds and incubated in 37 °C incubators for 30 min to produce hydrogels. The resulting hydrogels were aspirated and ejected out of a 23G needle attached to a syringe to produce anisotropic hydrogels. Sterilized filter paper was used to absorb excess moisture from hydrogels during the aspiration step. (**B**) Scanning electron microscopy showed that aspiration and ejection of hydrogels, collagen type I (Col), and Col laminin I (LN) resulted in hydrogels with aligned fibrils within. Scale bar = 2.5 μm. (**C**) Directionality histogram of aligned and non-aligned hydrogel images obtained from SEM data.

Col, Col HA, and Col LN hydrogels were produced as described in Section 2.1 but using larger molds (500 μL) to produce rectangular gels. Hydrogels with aligned collagen fibers were produced by aspirating the gel mixture into a blunted 23-gauge (G) needle (with an inner diameter of 0.31 mm) attached to a 10 mL syringe; slow and consistent pressure was applied (~15 min). A filter paper was placed on the opposite side of the needle (Figure 2) to slightly dehydrate the gel and aid in aspiration. Once the entire hydrogel was aspirated into the needle, the pulled hydrogel was then ejected out of the needle using a 5 mL syringe filled with 1× PBS solution. This method was previously used to produce highly dehydrated Col hydrogels [63].

### 2.5. Scanning Electron Microscopy and Image Analysis

The resulting hydrogels were thoroughly rinsed in deionized (DI) water, fixed in 4% paraformaldehyde (PFA) for 2 h at room temperature, and then dried by moving through an increasing concentration of ethanol (10 min in each solution) and hexamethyl disilazane before being further dried with a critical point dryer (~20 min) to maintain a collagen fibril structure. Samples were prepared for SEM by coating with a 10 nm gold/palladium coating. Images were taken for all groups of aligned and nonaligned gels on a JEOL JSM-700F Field Emission (JEOL USA, Peabody, MA, USA). To determine the direction of collagen fibrils, we used a plugin called “Directionality” available in Fiji (http://fiji.sc/User:JeanYvesTinevez) (accessed on 3 July 2024). This plugin is used to compute a histogram of the angular distribution that describes the number of structures in each direction using a typical Fourier-type analysis. To determine the fibril diameter size, SEM images obtained at 18–22 × 10^3^ magnification were opened in Fiji, and line measurement tools were used to determine the diameter size of 20 fibrils in each image. The average diameter length in nm and standard deviation were recorded.

### 2.6. Embryonic Spinal Cord Neuron Cultures

All animal work was carried out in accordance with appropriate guidelines from the University of Washington Institutional Animal Care and Use Committee (IACUC). Embryonic spinal cord neurons were isolated from embryonic day (E) 15 embryos, as described by Gingras et al., with slight modifications [64]. Briefly, pregnant mice were euthanized by intraperitoneal injections of 0.22 mL/kg Beuthanasia (Merck Animal Health, Summit, NJ, USA). Embryos were carefully removed and placed in cold isolation media containing Hanks’ Balanced Salt Solution (HBSS) (Thermo Fisher Scientific Inc., Waltham, MA, USA) with 1 mM HEPES (Thermo Fisher Scientific Inc.), 300 U/mL penicillin G, and 75 μg/mL of Gentamycin on ice. Embryos were decapitated, and spinal cords were removed from surrounding tissue under a dissecting microscope. Meninges were removed, and spinal cords were placed in fresh isolation medium, diced into 4–5 pieces, transferred to a 15 mL conical tube, rinsed twice with isolation medium, and allowed to settle. Spinal cords were then incubated in an enzyme solution containing L-15 medium without phenol red (Thermo Fisher Scientific Inc.) supplemented with 10 mM HEPES and 20–25 U/mL Papain (Worthington Biochemical Corp. Lakewood, NJ, USA) for 30 min. Tissue was gently triturated 5–10 times with fire-polished Pasteur pipettes until cell suspension was homogeneous, and then layered over a bovine serum albumin (BSA) cushion (4% wt/vol BSA in L-15 without phenol red) and centrifuged at 300× *g* for 10 min at 4 °C. Supernatant was removed and cells were gently re-suspended in cell culture medium containing Neuralbasal A (Thermo Fisher Scientific Inc.) supplemented with 2% B-27 (Thermo Fisher Scientific), 100 U/mL Penicillin G, 25 μg/mL Gentamycin, 0.5 mM GlutaMAX (Thermo Fisher Scientific Inc.), 10 ng/mL NT3, 10 ng/mL brain-derived neurotrophic factor (BDNF), 10 ng/mL glial-derived neurotrophic factor (GDNF), and 25 ng/mL ciliary neurotrophic factor (CNTF) (R&D systems, Minneapolis, MN). Cell solution was then gently layered over a Nycoprep gradient (Axis-Shield, Olso, Norway) with layers of 2 mL 8.01%, 2 mL 7.66%, and 2 mL 7.05% Nycoprep mixed with L-15 and centrifuged at 500× *g* for 20 min at 4 °C. Motor neurons were found at the interface of the 8.01%/7.66% layer and could be visualized as a white turbid band, while interneurons were pelleted at the bottom of the conical tube. Cells were selected out by gentle pipetting of layers, centrifuged at 100× *g* for 10 min at 4 °C, and then re-suspended in a cell culture medium.

Cell concentrations in hydrogels comprised approximately 30–40,000 cells/mL. Hydrogels with embedded cells were allowed to swell in a cell culture medium for approximately 1 h before pulling. Anisotropic gels with cells were obtained by aspiration and ejection through a needle (20G, Thermo Fisher Scientific Inc.) attached to 5 mL syringes. Samples were aspirated into the needle for 5–10 min each and then ejected by attaching the needle to a 1 mL syringe filled with a cell culture medium before ejecting the gel back into a well plate.

### 2.7. Immunocytochemistry

Samples were cultured for up to 14 days in vitro, and the resulting samples were fixed in 4% paraformaldehyde (PFA, Sigma-Aldrich) for 25 min and blocked with 3% donkey serum with 0.1% TritionX-100 for 1 h. The gels were then immuno-stained with rabbit anti-TUJ1 (ab18207, Abcam, Cambridge, MA, USA) or sheep anti-HB9 for spinal interneuron phenotypes (ab59795, Abcam) and chicken anti-GFAP for astrocytes and glia (ab4674, Abcam). Stained samples were examined, and photomicrographs were obtained using an Olympus FV1000 MPE BX61 multi-photon laser. Motor neurons embedded in hydrogels were immuno-stained with βIII Tubulin as described previously and imaged on an Olympus FV1000 MPE BX61 Multi-photon Microscope (Shinjuku, Tokyo, Japan). A FV10-MRG/R (495–540 nm, 575–630 nm) filter cube was used to image cells embedded in collagen hydrogels. Images were taken as z-stacks, and projections were produced using Fiji [65].

### 2.8. Spinal Cord Injury and Hydrogel Implantation

All rodent animal work was carried out after obtaining approval from the University of Washington Animal Care and Use Committee (IACUC # protocol #4362-01). Rats were anesthetized using isoflurane (5% to induce and 2.5% to maintain), and the area overlying the T7/T8 vertebrae was shaved, cleaned, and sterilized. An approximately 2.5 cm longitudinal incision centered over T7/T8 was made before subperiostal dissection of paraspinal muscles was carried out. A laminectomy was performed to expose the spinal cord at T7. To perform a dorsal hemisection, a thin scalpel was used to remove appx 1 mm^3^ of spinal tissue. Animals were randomly selected to be the following groups: SCI group with no implants (*N* = 6), SCI + non-aligned Col hydrogels (*N* = 8), and SCI + aligned Col hydrogel (*N* = 8). Hydrogels were kept sterile in 5 mg/mL gentamicin in PBS, and immediately before implantation, the hydrogels were trimmed (~1 mm in diameter; 1 mm in length) and placed directly onto the dorsal surface of the spinal cord. A small piece of adipose tissue was positioned on top of the hydrogel implant to reduce any dorsal tissue adherence to the hydrogel then the muscle and skin were sutured in layers to close the wound. Animals were treated with lactated Ringer’s solution (subcutaneous; 5–10 mL) and analgesics (buprenorphine; 0.03 mg/kg every 12 h for 48 h after surgery). Two animals did not survive the surgery, one from the non-aligned implant group and one from the aligned implant group. All remaining surviving animals were allowed to recover in warmed cages, and manual bladder expression was performed twice daily until normal void response recovered (up to 7 days post-injury). Animals were allowed to survive for 8 weeks post-injury (wpi).

### 2.9. Behavioral Analysis

In the weeks prior to surgery, the rats were all acclimated to the behavioral equipment. To examine their locomotion in an open-field area, the Basso, Beattie, Bresnahan (BBB) locomotor rating scale was performed one week before the injury and two weeks post-injury. The BBB test is a sensitive, observer-scored test of locomotor behavior [66,67]. The animals were placed into a ~100 cm diameter arena with 19 cm walls with a lightly textured floor. For four minutes, each animal was permitted to explore the arena while two blinded observers recorded the locomotor actions according to the BBB locomotor rating scale. The test was also video recorded for verification at later timepoints.

Additionally, CatWalk XT (Noldus Information Technology Inc., Leesburg, VA, USA)—a gait analysis system, was used to assess the motor function and coordination of the rats. This test comprises a large rectangular pathway that serves as an illuminated glass platform, and data are collected from below the platform through a video camera. Gait related parameters—such as stride pattern, individual paw swing speed, stance duration, and pressure can be obtained from each run from the rat. Baseline data of 10 runs per animal was collected prior to surgery, and each week post-surgery to 8 weeks total, 10 runs were collected per animal. All data were saved and analyzed using CatWalk XT software.

### 2.10. Histological Analysis of Spinal Cord Tissue

At the time of sacrifice, rats were euthanized by intraperitoneal injection of 0.8 mL of Beuthanasia–D (390 mg/mL pentobarbital sodium and 50 mg/phenytoin sodium, Merck Animal Health), followed by transcardial perfusion with 250 mL of cold PBS and 250 mL of cold 4% paraformaldehyde. Tissue samples were dissected out and post-fixed in 4% paraformaldehyde for 12–16 h at 4 °C. Samples were then cryo-protected by placing them in 10, 20 and 30% sucrose solutions and stored in 30% sucrose solution until use. Standard immunohistochemistry procedures were performed using primary antibodies to detect neurons (Pan Neuronal antibody [1:500, MAB 2300, Millipore, Burlington, MA, USA], or Beta III tubulin [1:1000, Ab78078, Abcam]) and astroglia cells (GFAP [1:1000; Z00034, DAKO, Glostrup, Denmark]). To determine the extent of alignment of processes from cultured neurons within the hydrogel, we used the “Directionality” tool in Fiji (https://imagej.net), as previously described [68,69].

### 2.11. Statistics

To determine the alignment of neurite outgrowth in vitro and neurites in vivo, coherency and alignment data were analyzed with a two-tailed Student’s t-test to compare the coherency of neurites within aligned and non-aligned hydrogels (both Col and Col LN gels), and significance was set at *p* = 0.05. Either one-way or two-way analysis of variance (ANOVA) with Tukey’s post hoc analysis was used to analyze behavioral studies to examine the effects of lesion status alone or lesion status with time after injury with the significance level set at *p* = 0.05.

## 3. Results

Thermally gelling hydrogels were produced using type I collagen (Col) at 1.5 mg/mL concentration. In addition, interpenetrating networks of hydrogels containing hyaluronic acid (HA; 1.5 mg/mL) and laminin (LN; 1.5 mg/mL) were also produced (Figure 1A). To estimate gelation times, light absorption of the pre-gel solution within 96 well plates was examined using a spectrophotometer at 490 nm. It has previously been shown that the optical density of a protein in solution is proportional to the state of protein aggregation of the material [47,70,71]. We found that Col solution formed gels faster (T_95_ = 17 min) compared to Col LN and Col HA solutions, with T_95_ at 22 min (Figure 1B). We also examined the bulk compressive moduli of these hydrogels and found that Col gel had the highest compressive moduli (1290 ± 94 Pa) compared to Col HA (1040 ± 104 Pa) and Col LN (1008 ± 73 Pa) (Figure 1C), and there was no statistical difference in the compressive modulus between all three of the hydrogels examined. It is important to note that all compressive moduli detected in the three hydrogels were softer than the reported compressive modulus of ~9 kPa for adult rodent spinal cord tissue [62]. In a previous study, we measured the storage and loss moduli of these three gel types (Col, Col LN, and Col HA) and showed that Col HA had the lowest shear moduli while the Col LN hydrogels had the highest [47].

### 3.1. Producing Alignment Within Hydrogels

To introduce aligned topography within amorphous hydrogels, we applied the aspiration and ejection method (Figure 2). This method has been used previously to produce aligned morphology in collage-based hydrogels at higher concentrations (2.1 mg/mL) [63]. Here, we showed that this technique of aspiration ejection can be used (1) at a lower concentration (1.5 mg/mL) without breaking the structural integrity of the hydrogels and (2) with interpenetrating networks of hydrogels (Col LN) to produce aligned topography within these hydrogels. The effects of the aspiration–ejection method on Col and Col LN gels were examined using SEM. Photomicrographs in Figure 2B show that the aspiration–ejection method resulted in a dramatic directional organization of fibrils within the gels. Aligned fibrils were seen within Col hydrogels and Col LN hydrogels (Figure 2B). The fibril size for non-aligned and aligned Col were 101 ± 25 nm and 136 ± 29 nm, respectively. These diameters are consistent with the typical size range for collagen fibrils seen in the literature (50–250 nm) [72]. The fibril size for non-aligned and aligned Col LN gels were 123 ± 15 nm and 124 ± 21 nm, respectively.

### 3.2. Embryonic Spinal Cord Neurons in Aligned Scaffolds

**Figure 3:**
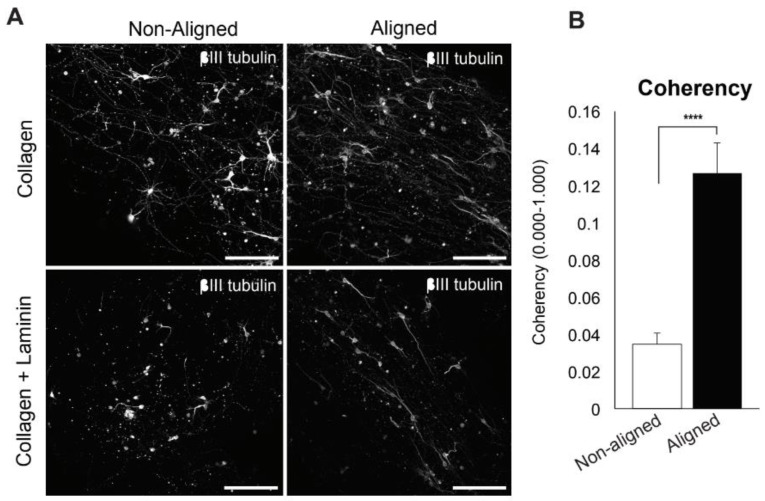
Anisotropic hydrogels with cells can support and guide the neurite outgrowth of embryonic spinal neurons in vitro. (**A**) Coherency was used as a relative measure of neurites and their growth direction. Neurons within anisotropic and aligned hydrogels (both collagen type I (Col) and Col laminin I (LN) gels) produced neurites with significantly more coherent morphology than cells within non-aligned hydrogels. (**B**) Photomicrographs were taken using a two-photo microscope of spinal neurons labeled with βIII Tubulin (a neuronal marker) at 7 days in vitro in non-aligned and aligned Col and Col LN gels. Scale bar = 200 μm. Two-tailed student t-test showed significantly higher coherency of neurites in aligned hydrogels compared to the ones on non-aligned hydrogels; **** *p* < 0.0001.

Next, we examined whether primary neuronal cells can be encapsulated and cultured within aligned hydrogels. In this portion of the study, we only used Col, Col LN, and Col HA hydrogels. The aspiration–ejection technique was used to align Col and Col LN hydrogels containing primary E14 spinal neurons. We then cultured the resulting condensed hydrogels with cells for 10–14 days in culture. We found that cell growth and differentiation within Col HA were poor. This could be due to the fact that the presence of high molecular weight HA has been shown to keep cells in an undifferentiated state [73,74], and the mature spinal cord contains very little HA [75]. Both dissociated motor neurons and interneurons survived in aligned Col and Col LN hydrogels and exhibited processes with aligned morphology, while neurons within non-aligned hydrogels showed directionally haphazard growth (Figure 3). This suggests that the orientation of the fibrils within the hydrogel directs the preference for neurite outgrowth. The degree of alignment was calculated using a coherency test, which confirmed that neurites within the aligned hydrogels were significantly more coherent, i.e., aligned, compared to neurons within non-aligned hydrogels (*p* < 0.0001). Oligodendrocytes and astrocytes were examined for alignment to scaffolds, but we found that there were very few astrocytes and oligodendrocytes, and they did not show preferential process outgrowth.

### 3.3. Functional Evaluation

**Figure 4:**
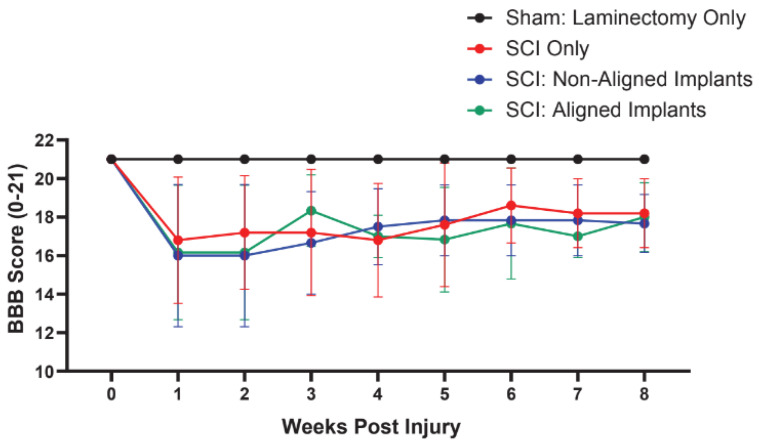
Basso, Beattie, and Bresnahan (BBB) open-field locomotor test for animals with spinal cord injury (SCI) alone, SCI + non-aligned Col gel implants, or SCI + aligned Col implants each week to 8 weeks post-injury. Analysis by two-way ANOVA (lesion status and time) followed by Tukey’s post hoc test showed that all lesioned groups recovered similarly over time.

We next implanted non-aligned and aligned Col hydrogels in vivo after a dorsal hemisection surgery in rats. After the hemisection at T7/T8, we placed either non-aligned Col gel (SCI + non-aligned) or aligned Col gel (SCI + aligned) onto the dorsal surface of the injury site. Animals in the SCI-only group received a hemisection injury, but no hydrogels were implanted. We performed behavioral analysis using the BBB test to evaluate the open-field locomotor movements of animals before and after injury with and without hydrogel implantation (Figure 4). A two-way ANOVA showed that there was a significant interaction between lesion status and time (*p* > 0.05). As expected, there was a significant effect of injury (F(3, 18) = [5.04], (*p* = 0.01)), and time after injury (F(3.75, 67.49) = [10.78], *p* < 0.0001)). Additionally, we found that there was no statistical difference between any of the SCI animals regardless of implant type. At 8 weeks post-injury, animals with SCI + dural opening, SCI + non-aligned Col gel implant, and SCI + aligned gel implant had BBB scores of 17.2, 16.0, and 15.8, respectively.

**Figure 5:**
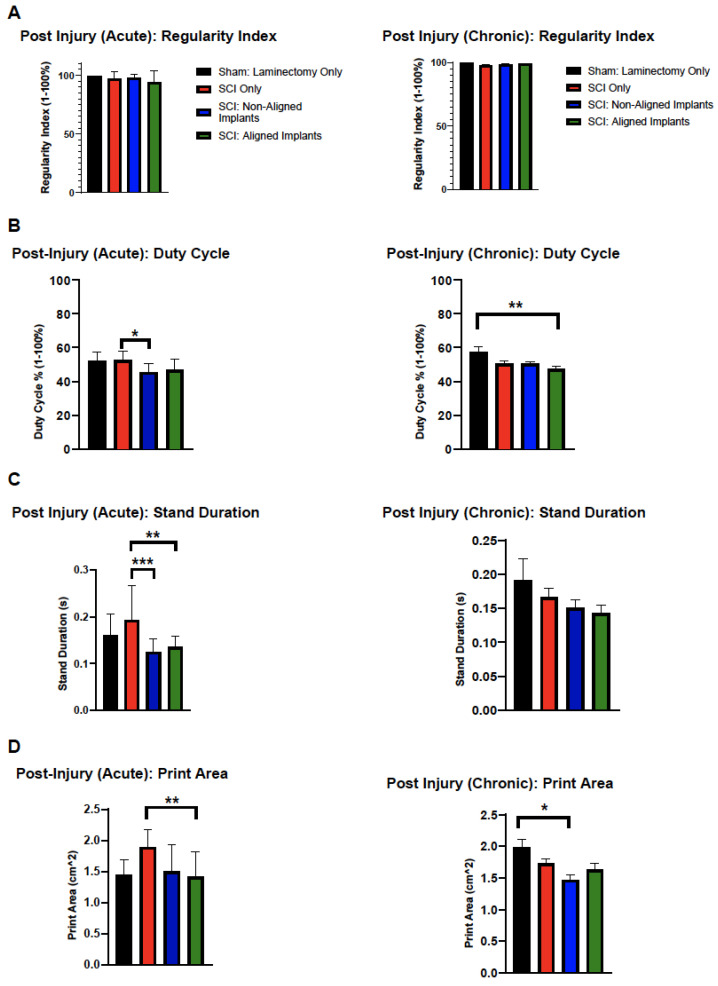
Catwalk analysis of regularity index, duty cycle, stand duration, and the print area between groups at acute (1–3 weeks post-injury) and chronic timepoints (6–8 weeks post-injury). (**A**) Regularity index describing the exclusive use of normal step sequence patterns during uninterrupted locomotion. (**B**) Duty cycle (%) is the ratio of stand time to step cycle (duty cycle = stand time/step cycle). (**C**) Stand duration (s) calculated by the duration of contact with the walkway. (**D**) The print area is the measure of the area of a step-in cm^2^. Analysis by one-way ANOVA followed by Tukey’s post hoc test; * *p* < 0.05, ** *p* < 0.01, *** *p* < 0.001.

Catwalk analysis was divided into acute and chronic data, where acute was defined to be weeks 1–3 post-injury, whereas chronic was defined as weeks 6–8 post-injury. A two-way ANOVA was carried out to detect statistical significance between lesion status and time after injury. The regularity index showed no statistical significance between all groups examined and all timepoints (*p* > 0.05, Figure 5A).

In analyzing duty cycle, which is the measure of stance duration as the percentage of step cycle duration, there were significant effects of treatment (F(3, 152) = [14.43], (*p* < 0.0001)) as well as of time after injury (F(8, 152) = [13.43], (*p* < 0.0001)) (Figure 5B). In the acute condition, there was also a significant reduction between the SCI only and the SCI with non-aligned implants (*p* < 0.05), with an average value of 52.77% and 45.51%, respectively. In the chronic condition, laminectomy-only and SCI + aligned implants had a significantly lower percent of duty cycle compared to laminectomy-only animals (*p* < 0.01; mean values of 48.36% and 50.39%, respectively).

For stand duration, the duration of contact with the walkway, there were significant effects of treatment (F(3, 152) = [11.11], (*p* < 0.0001)) and of time after injury (F(8, 152) = [8.92], (*p* < 0.0001)) (Figure 5C). At acute timepoints, there was a significant reduction in stand duration between SCI only (0.1929 s) and SCI with non-aligned (0.1144 s) as well as SCI only and SCI aligned groups (0.1380 s) with *p* < 0.01 and *p* < 0.01, respectively. At chronic timepoints, there was no statistical significance in stand duration between all treatment groups.

Lastly, we examined the print area and found that there was the size of each footprint, there were significant effects of treatment (F(3, 152) = [9.81], (*p* < 0.0001)) and of time after injury (F(8, 152) = [14.58], (*p* < 0.0001)). (Figure 5D) At acute timepoints, there was a statistical significance between SCI only and SCI aligned (*p* < 0.01) with print area values of 1.890 cm^2^ and 1.305 cm^2,^ respectively. In the chronic timepoint, there was a significant difference between sham and SCI non-aligned (*p* < 0.05) and values of ~2 cm^2^ and 1.4 cm^2,^ respectively.

### 3.4. Histological Analysis

**Figure 6:**
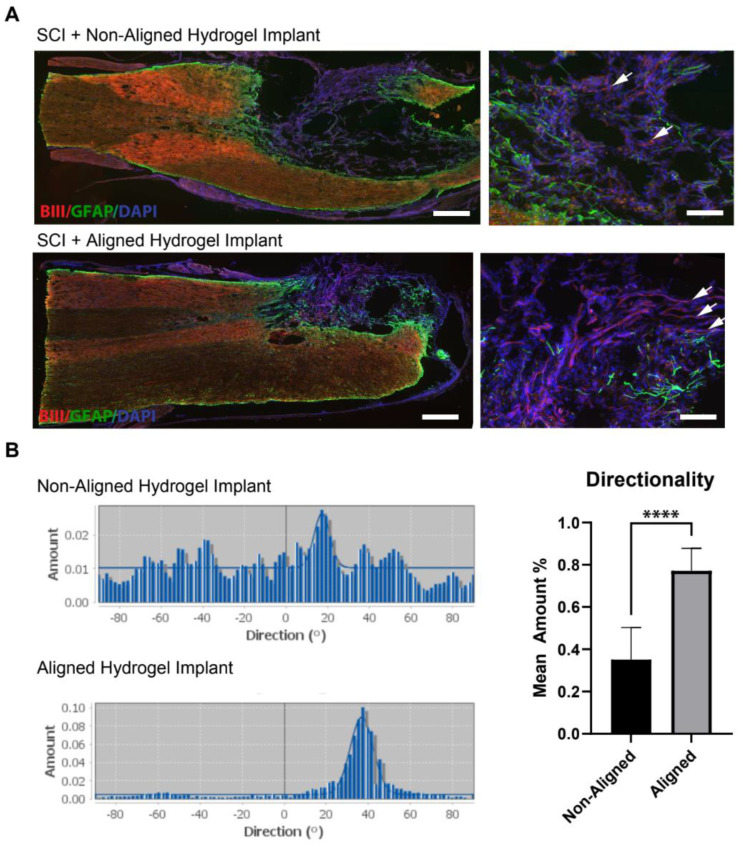
Anisotropic aligned collagen type I (Col) gels can support axonal growth after acute spinal cord injury. Non-aligned and aligned Col gels were implanted acutely after a bilateral dorsal hemisection of the thoracic spinal cord at T7/T8. The dural lining was then opened, and the hydrogels were trimmed (~1 mm in diameter; 1 mm in length) and implanted into the injury site. (**A**) At 8 weeks post-injury, spinal cord sections were collected and examined histologically. Astrocytes (glial fibrillary acidic protein [GFAP]) staining showed an astrocytic response within the spinal cord parenchyma (detected using GFAP, green). Neurons and neurites were detected using anti-Beta III Tubulin antibody (axons, red). Scale bar = 500 μm. Higher magnification image of smaller areas (right panel) showing both non-aligned and aligned neurites within anisotropic Col hydrogels (white arrows). Scale bar = 50 μm. (**B**) Directionality histogram of Beta III Tubulin staining in vivo from aligned and non-aligned hydrogel implants. Example of directionality histogram within aligned and non-aligned gels, showing a well-fitted curve within aligned, peaking around 40 °C. Comparison of mean amounts across both aligned and non-aligned; two-tailed test, **** *p* < 0.0001.

Histological examination of spinal cord tissue after 8 weeks in vivo revealed that both non-aligned and aligned hydrogels supported the growth of neurites within the hydrogels (Figure 6A). Importantly, the axons within the aligned hydrogels grew parallel to the implanted hydrogels and the longitudinal axis of the cord (Figure 6B).

To quantify the alignment of the axon fibers, the “Directionality” tool in Fiji was used to capture three subsections of each image of Beta III Tubulin-stained sections from aligned and non-aligned hydrogel implants from aligned (*N* = 3) and non-aligned (*N* = 4) animals. The mean amount—indicating the percent of structures that align in the direction of the highest peak—is displayed in Figure 4. Statistical significance (*p* < 0.0001) was found between the groups. Directionality histograms were then created for both groups, which display a well-fitted curve within aligned gels peaking at around 40 °C.

## 4. Discussion

Spinal cord injury remains a serious clinical challenge, and very little progress has been achieved in terms of therapeutic treatments. It has long been suggested that a combinatorial approach for repairing the spinal cord could be the most effective in returning more finely tuned limb movements. Hydrogels, including the ones made from natural-based polymers, are ideal biomaterials and have been developed to serve as scaffolds and/or carriers for cells, proteins, or small molecule therapeutics [6,36]. In this study, we present a technique utilizing a Col-based thermally gelling 3D hydrogel system to generate aligned fibrils. This was accomplished using a simple aspiration–ejection system developed previously [63]. We extended the previous work by including other neural ECM molecules (LN, HA) to better mimic the neural ECM. We found that the Col fibrils can be aligned using the aspiration–ejection method and that these co-gel systems can support the alignment of neural elements both in vitro and in vivo. Our data support the use of Col-based hydrogels in combination with the ECM component LN, in promoting directional neurite growth both in vitro and in a pre-clinical model of spinal contusion injury.

In vitro studies show that embryonic spinal motor neurons and interneurons exhibited good survival and neurite outgrowth aligned to collagen fibrils in the Col LN group but not in the Col HA group. HA is normally not cell adhesive, and the presence of either other cell adhesive ECM molecules such as Col, LN, or cell adhesive peptides are needed to support cell adherence and survival in HA-based hydrogels [76]. Other groups report neuron attachment and neurite extension on modified HA-based gels [77,78]. However, once implanted into the spinal cord after injury model, HA-based hydrogels did not enhance axonal growth [79]. In our experiments, dissociated embryonic spinal cord cells had low survival in Col HA matrices compared to Col alone or Col LN gels regardless of alignment, and the few neurons attached had limited process outgrowth. Other groups have reported similar difficulty in neuronal cell adherence to HA hydrogels [80]. Presumably, differences in protocols concerning concentration, molecular weight, type of HA, and cell types used could influence cell attachment and survival. Using aligned gels (Col and Col LN), embryonic spinal motor neurons aligned to the fibrils within the hydrogel such that it was extremely rare to find cells in the Col LN group that were not aligned to the direction of the scaffold.

We also examined the presence of oligodendrocytes and astrocytes at 14 days in vitro but did not see many astrocytes or oligodendrocytes developed within any of the hydrogels examined. Astrocytes are typically not generated in high numbers until later stages of development (E19–P3) [81]; thus, the low cell count and alignment of labeled astrocytes are not surprising, as the spinal neurons we used were isolated at E14. Future studies examining the development of astrocyte and oligodendrocyte precursors within these scaffolds would be of interest, as both cell types play significant roles in the recovery from SCI. Astrocytes play many crucial roles in neuronal support, including glutamate uptake, structural support, metabolic control [82,83], synaptic transmission, and regulation of the neuropil [84]. During traumatic injury, microglia and reactive astrocytes result in cell death of neurons and oligodendrocytes and glial scar formation [85,86]. Interestingly, we have shown previously that Col LN HA hydrogels can support the growth and preferential differentiation of oligodendrocytes from spinal progenitor cells after SCI, resulting in better functional recovery in a rodent contusion model [47]. Thus, future studies examining the differentiation of both immature astrocytes and oligodendrocytes within these hydrogels will be of great interest and are likely to be critical for a successful regenerative strategy after SCI. In addition to incorporating cells, these hydrogels can also be combined with a variety of different growth factors and chemical cues, thus having a wide range of applications in nerve regeneration and repair.

The in vivo implantation of non-aligned and aligned Col gel showed that after eight weeks, endogenous neurons extended many neurites into the hydrogels. Aligned neurofilament-positive fibers were seen within aligned Col hydrogels, suggesting that the topographical cues within the hydrogels can guide the direction of neurite outgrowth in vivo. Examination of the glial response showed that there was no significant difference between all the experimental groups examined. Previous work from our lab demonstrated that the application of HA-containing hydrogels resulted in decreased scar formation compared to control animals after SCI [47,76]. We posit that the decrease in scar formation/thickness (GFAP-positive) in the previous studies is due to the presence of high molecular weight HA present within the hydrogels. In assessing hind limb recovery before and after injury in all behavioral assessments, we notice a significant motor impairment at week 1 vs. week 0 (Figure 4). At 8 weeks post-injury, we did not detect any significant recovery of hind limb function in either hydrogel-implanted group. These findings were not surprising since we did not include treatment to limit the glial scar, which is a known inhibitor of neurite outgrowth [27,87], and therefore did not expect significant axonal growth back into the host’s spinal cord. This is a limitation of our study, and future studies are needed to confirm whether aligned hydrogels engineered using natural ECM components can be combined with scar-inhibiting reagents to improve functional recovery after SCI. The addition of compounds in conjunction with hydrogels such as chondroitinase ABC (chABC), which have been proven to promote the sprouting of axons into lesion sites and increase function recovery, may be the next step to observe significant functional improvement.

In future studies, it will be important to examine whether aligned Col-based hydrogels can significantly increase the rate of neurite outgrowth, promote functional connections distal to the site of injury, and if they can support oligodendrocyte growth to generate myelinated fibers. After the injury, even neurons that show plasticity and the ability to grow neurites have a limited time window where regenerative programs are active [7]. Therefore, it will be advantageous to utilize scaffolds that can support faster neurite outgrowth and migration of cells, which can significantly impact the extent of recovery after SCI.

## 5. Conclusions

This study reports the development and use of a simple aspiration–ejection system to produce aligned topography within hydrogels made from the biomimetic thermally gelling polymer collagen. All hydrogels examined (Col, Col LN, and Col HA) can form gels at 37 °C in 30 min and have mechanical properties similar to that of spinal cord tissue. Both aligned and non-aligned Col and Col LN hydrogels were able to support the encapsulation of embryonic spinal motor and interneurons. After 2 weeks in culture, neurons within the aligned hydrogel contained numerous processes that were aligned compared to non-aligned hydrogels, suggesting that aligned topography within the hydrogels can guide neurite outgrowth. The in vivo implantation of Col gels showed that these hydrogels integrate well into the host tissue and support/guide endogenous axonal growth. Future studies are needed to examine whether the implantation of anisotropic Col gels can support significant regeneration of endogenous axons to result in improved functional recovery in experimental models of SCI.

## Data Availability

Data included in this manuscript will be available through the Open Data Commons for Spinal Cord Injury (ODC-SCI) (https://odc-sci.org/about/about-us) after publication.
